# Ten year survival after excision of squamous cell cancer in Zenker's diverticulum: report of a case

**DOI:** 10.1186/1477-7819-4-17

**Published:** 2006-03-28

**Authors:** Gianlorenzo Dionigi, Fausto Sessa, Francesca Rovera, Luigi Boni, Gianpaolo Carrafiello, Renzo Dionigi

**Affiliations:** 1 Department of Surgical Sciences, University of Unsubria, Varese, Italy; 2 Department of Clinical and Biological Sciences, University of Insubria, Varese, Italy; 3 Department of Diagnostic and Interventional Radiology, University of Unsubria, Varese, Italy

## Abstract

**Background:**

Zenker's diverticulum (ZD) has been increasingly recognized as a site of primary epithelial malignancy. Pitt in 1896 described the first case.

**Methods:**

Between 1990 and 2005, 30 patients affected of esophageal diverticulum were referred to our Department.

**Results:**

The pathological results revealed one case of squamous cell carcinoma. On follow-up 10 years after diverticulectomy alone, the patient was alive and well without evidence of recurrence.

**Conclusion:**

Our case reported provides additional data on clinical decision when the tumor is well localized without full-thickness penetration or extension to the line of resection. In this patient, long-term survival and apparent disease control have been effected by diverticulectomy alone. A case of such long survival is very rare.

## Background

Pharyngoesophageal diverticulum was first described by Ludlow, who observed a "preternatural pocket" in the cervical esophagus at autopsy [1]. It is the most common type of diverticulum, with a prevalence of 0.01–0.11% in the general population and an incidence of 1.8–2.3% in patients complaining of dysphagia [2]. Zenker and von Ziemssen first described the pathogenesis of the diverticulum [3]. In recent decades, Zenker's diverticulum (ZD) has been increasingly recognized as a site for squamous cell carcinoma. Pitt described the first case in a 50-year-old [4]. In a review of squamous cell carcinoma reported, about 40 cases could be identified up to 2000 [5].

## Case presentation

**Figure 1:**
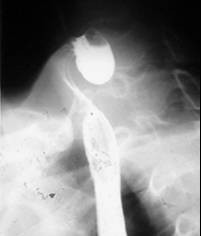
Radiological stage III diverticulum.

**Figure 2:**
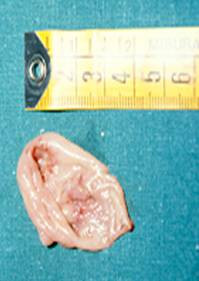
Postoperative diverticulum specimen.

**Figure 3:**
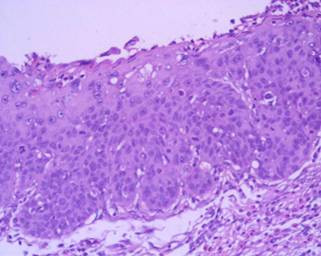
Photomicrograph showing Infiltrating squamous cell carcinoma (hematoxylin and eosin × 40).

**Figure 4:**
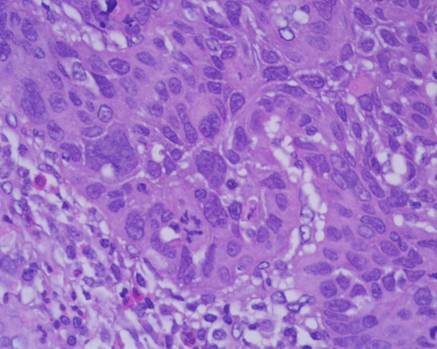
Photomicrograph showing area of severe dysplasia (hematoxylin and eosin × 100).

In a 15-year period, between 1990 and 2005, 30 patients affected of Zenker's diverticulum were referred to our Department. Histopathological examination of all specimens after surgery was performed using standard staining techniques. The pathologic results revealed diverticulitis in 23/30 cases and one case of associated squamous cell carcinoma arising in the lining of the diverticulum in a 75-year-old Caucasian man, who was seen in May 1995. He had a history of heavy smoking (80 pack/year) and alcohol consumption. He was referred with a barium esophagogram demonstrating a large wide-mouthed diverticulum of the cervical esophagus of 5 × 5 cm extending to the left with barium passing into the cervical and thoracic esophagus without spilling of into the tracheobronchial tree (Figure 1). The patient admitted to having occasional episodes of dysphagia, regurgitation, and sialorrhea in the past 2 months. Further work-up of his diverticulum included an endoscopy that confirmed the diverticulum without irregularity of the mucosa in the esophageal inlet but with diffuse edema of the entire hypopharangeal mucosa. He underwent left transcervical one-stage pharyngoesophageal diverticulectomy with removal of the sac using a stapling device (TA-30) and myotomy. Pathological examination revealed a 6 × 5 × 1.5 cm sac with normal external appearance (Figure 2). The sac contained an epithelial lining with stratified squamous epithelium. The submucosa showed fibrous tissue surrounding it with non-specific chronic mucosal inflammation noted in the wall of the diverticulum. Nearer the neck of the sac, scanty muscle fibers were found. An ulcerated tumor (grade 2, squamous cell carcinoma) measuring < 1 cm in diameter was identified at the fundus of the diverticular sac. The lesion had micro invasion of the submucosa and the muscularis mucosa (pT2) (Figure 3). Marked epithelia dysplasia was noted surrounding the lesion (Figure 4). Intense peri-tumoral lymphocyte reaction was present (Figure 3, 4). The mucosa at the site of excision was free of involvement (R0). Because of the patient's age, the nature of the neoplasm encountered, and its localization remote from the line of resection, no further treatment was advised. Follow-up assessments included every 4 months for the first year, every 6 months for the next 2 years, and annually thereafter-physical exams, x-rays, imaging studies and endoscopy. Between scheduled appointments, patient reported any health problems to GP as soon as they appeared. On follow-up in June 2005, 10 years after diverticulectomy alone, the patient was alive without esophageal symptoms or evidence of recurrence.

## Discussion

Certain unique features highly suggest an additional diagnosis of associated carcinoma in a diverticulum, as a sudden increase in the severity of symptoms, particularly progressive dysphagia or aphagia, cervical pain, more marked regurgitation of food with bloody mucus, rapid weight loss, and hematemesis. Furthermore, cigarette smoking, previous aero-digestive tract malignancy and prolonged history of a ZD have been identified as risk factors for the development of cancer [5]. Chronic irritation and inflammation in these large diverticular pouches secondary to food retention, are probably contributing factors in the development of carcinoma. In our case the pathological examination of the specimen revealed dysplasia surrounding the tumor: we would speculate that in many patients who developed an invasive cancer in ZD, the lesion would have gone through a period of intraepithelial disease prior to the development of invasive tumor. In associated cancer cases, the diagnosis is often delayed [6]. Irregularity of the lining of the diverticulum, or of the pharyngeal or esophageal wall raises the possibility of tumor arising in the diverticulum itself. Patients with pharyngoesophageal diverticulum should undergo careful endoscopic evaluation before any definitive form of surgery [7]. Surrounding edema can prevent radiological and endoscopic diagnosis in patients as happened in our patient and small invasive carcinoma was detected only at histopathologic examination of the pouch after excision. The question about right treatment of Zenker's diverticulum is interesting and important. Diverticulectomy is nowadays a much-discussed treatment because of increased frequency of complications and longer postoperative stay compared to diverticulopexy and endoscopic diverticulectomy [6]. Complications of external approach compared to endoscopic treatment are opposed by the risk of unnoticed development of malignancy in endoscopic treatment. The risk of malignancy seems to be low; but the real population based incidence is not yet known. It is for this reason that Authors proposed that patients less than 65 years and who have a large pouch should undergo excision of the pouch and pathological examination of the pouch [8,9]. We did encounter carcinoma arising within a diverticulum in one patient. On follow-up in June 2005, 10 years after diverticulectomy, the patient was alive and well without evidence of recurrence. Huang reported on two patients (one carcinoma *in-situ*, the second with invasive cancer) who had simple diverticulectomy with clear resection margins: the patients were free of disease after 8 and 4-year respectively [10]. In this patient, long-term survival (ten years) and apparent disease control have been effected by diverticulectomy alone.

## Competing interests

The author(s) declare that they have no competing interest.

## Authors' contributions

**GD**: Acquisition of data, drafting of manuscript

**FR, GC**: Study conception and design

**LB, FS**: Analysis and interpretation of data

**RD**: critical revision and supervision
